# Computational Study of Catalytic Poisoning Mechanisms in Polypropylene Polymerization: The Impact of Dimethylamine and Diethylamine on the Deactivation of Ziegler–Natta Catalysts and Co-Catalysts

**DOI:** 10.3390/polym17131834

**Published:** 2025-06-30

**Authors:** Joaquín Alejandro Hernández Fernández, Katherine Liset Ortiz Paternina, Heidis Cano-Cuadro

**Affiliations:** 1 Chemistry Program, Department of Natural and Exact Sciences, San Pablo Campus, Universidad de Cartagena, Cartagena de Indias D.T. y C., Cartagena 130015, Colombia; 2 Department of Natural and Exact Science, Universidad de la Costa, Barranquilla 080002, Colombia; 3 Grupo de Investigación GIA, Fundacion Universitaria Tecnologico Comfenalco, Cr 44 D N 30A, 91, Cartagena 130015, Colombia; 4 Department of Civil and Environmental, Universidad De La Costa, Barranquilla 080002, Colombia

**Keywords:** density functional theory, catalyst, cocatalyst, dimethylamine, diethylamine, polymerization, Ziegler–Natta

## Abstract

In this study, density functional theory (DFT) was used to analyze the processes that govern the interactions among triethylaluminum (TEAL), the Ziegler–Natta (ZN) catalyst, and the inhibitory compounds dimethylamine (DMA) and diethylamine (DEA) during olefin polymerization. The structural and charge characteristics of these inhibitors were examined through steric maps and DFT calculations. Combined DFT calculations (D3-B3LYP/6-311++G(d,p)) and IR spectroscopic analysis show that the most efficient way to deactivate the ZN catalyst is via the initial formation of the TEAL·DMA complex. This step has a kinetic barrier of only 27 kcal mol^−1^ and a negative ΔG, in stark contrast to the >120 kcal mol^−1^ required to form TEAL·DEA. Once generated, TEAL·DMA adsorbs onto the TiCl_4_/MgCl_2_ cluster with adsorption energies of −22.9 kcal mol^−1^ in the gas phase and −25.4 kcal mol^−1^ in n-hexane (SMD model), values 5–10 kcal mol^−1^ more favorable than those for TEAL·DEA. This explains why, although dimethylamine is present at only 140 ppm, its impact on productivity (−19.6%) is practically identical to that produced by 170 ppm of diethylamine (−20%). The persistence of the ν(Al–N) band at ~615 cm^−1^, along with a >30% decrease in the Al–C/Ti–C bands between 500 and 900 cm^−1^, the downward shift of the N–H stretch from ~3300 to 3200 cm^−1^, and the +15 cm^−1^ increase in ν(C–N) confirm Al←N coordination and blockage of alkyl transfer, establishing the TEAL·DMA → ZN pathway as the dominant catalytic poisoning mechanism.

## 1. Introduction

**Figure 1:**
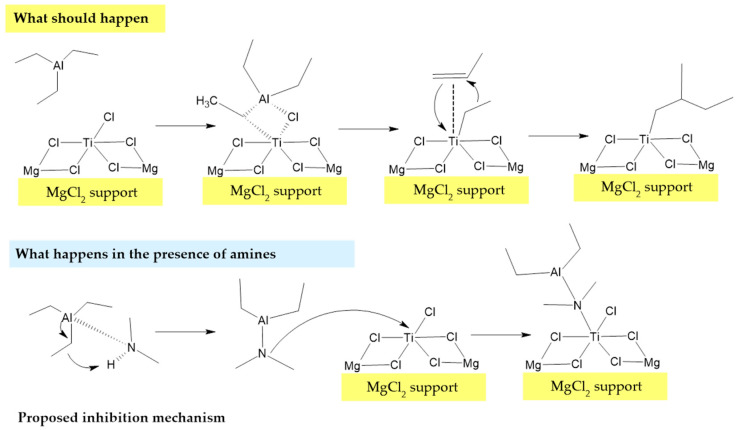
Proposed inhibition mechanism for the Ziegler–Natta system in the presence of dimethylamine.

The poisoning of Ziegler–Natta (ZN) catalysts is a critical phenomenon that significantly impacts efficiency and selectivity in polyolefin production. This process involves the interaction of chemical species with the active sites of the catalyst, reducing its activity [1,2]. Studying selective poisoning is essential to understand how certain electron donors can alter the catalyst’s properties, both in terms of activation and deactivation [3,4,5,6,7] (Figure 1). From research reported in the literature, it can be evidenced that the reduction of active sites can be caused by poisoning [8,9], and this also affects the propagation speed of the chains during the propagation process [10,11]. These activation and deactivation processes are also affected by the coordination of electron donors in the active sites. It is important to emphasize that the mechanisms by which these processes occur have not yet been fully elucidated, thus indicating that scientific research still needs to be explored and especially those related to kinetic aspects [4].

Since their discovery in the 1950s, highly active MgCl_2_-based Ziegler–Natta catalysts have played a key role in global polyolefin production and have been used in most industrial processes over the past decades. Given the importance of the applications of polyethylene and polypropylene, it is necessary to increase research efforts to improve the performance of Ziegler–Natta catalysts and some chemical agents or electron donors that improve their activity and stereospecificity [3,12,13,14,15]. Their widespread use is attributed to the combination of a low-cost polymerization process with outstanding catalytic activity, high stereoselectivity [16], and effective control of molecular weight [17,18]. Notably, Al-alkyl species have a significant impact on the stereoselectivity of propylene polymerization and the determination of molecular mass. These compounds exhibit high reactivity when exposed to most organic and inorganic substances, with notable exceptions including noble gases, saturated and aromatic hydrocarbons, and alkynes [19,20,21].

Given the impact of aluminum alkyl compounds on stereoisomeric selectivity and molecular weight, it is logical to broaden the discussion to triethylaluminum (TEAL), a key component of the ZN system. Numerous studies have shown that TEAL not only alkylates and reduces Ti sites but also scavenges impurities; however, its strong affinity for small Lewis bases makes it susceptible to poisons such as alcohols, ketones, and—most notably—amines. In the 1980s, Sacchi and co-workers demonstrated that amines can exert two simultaneous effects: (i) deactivate both isotactic and atactic sites and (ii) modulate the distribution of active sites through the formation of Al–N complexes [6].

Even trace amounts of polar compounds lower the catalyst’s efficiency and stereoselective performance [22]. Hernández-Fernández et al. observed productivity losses of up to 20% in polypropylene production when minute quantities of propanol or arsine were introduced, highlighting the extreme sensitivity of the ZN system to strong nucleophiles [23]. Similarly, Tangjituabun et al. ranked the deactivating power of several functional groups (methanol > acetone > ethyl acetate) and linked this trend to the ease of forming inactive Ti–X species [4].

Aliphatic amines stand out among these “inhibitors” because they can form 1:1 adducts with TEAL that subsequently migrate to the MgCl_2_/TiCl_4_ surface, blocking monomer insertion. Recent spectroscopic studies and DFT calculations show that, after TEAL activation, most Ti^4+^ sites are reduced to Ti^3+^ and become bonded to Al-alkyl fragments; the presence of a strong base disrupts this equilibrium, creating “dead” sites incapable of further chain growth [24].

Despite numerous investigations into the structure, activation, polymerization behavior, and polymer properties of ZN catalysts [25,26,27,28], their reactions with inhibitors have received far less attention [29]. The magnitude and nature of the interactions between poisons and the various components of the ZN process, therefore, remain largely unquantified. This work focuses specifically on the dual interaction amine ⇄ TEAL ⇄ catalyst. Using DFT simulations, we explore (i) the formation of TEAL·amine adducts and their kinetic feasibility; (ii) the stability of these assemblies once adsorbed on a TiCl_4_·(MgCl_2_)_n_ surface; and (iii) the correlation between binding energies and the experimentally observed productivity losses for dimethylamine and diethylamine. By filling this gap in the literature on amine-mediated poisoning of TEAL—and consequently of ZN—our study provides an atomistic foundation for designing effective mitigation strategies.

## 2. Materials and Methods

### 2.1. Computational Study

In this investigation, we used the Gaussian 16 software Rev. A.03, (GGA) B3LYP with the correction factor D3 and (6-311++G(d,p)) [30,31,32], and the UCSF ChimeraX package version 1.8 [33]. We calculated the frequency and analyzed the intrinsic reaction coordinates. 

**Figure 2:**
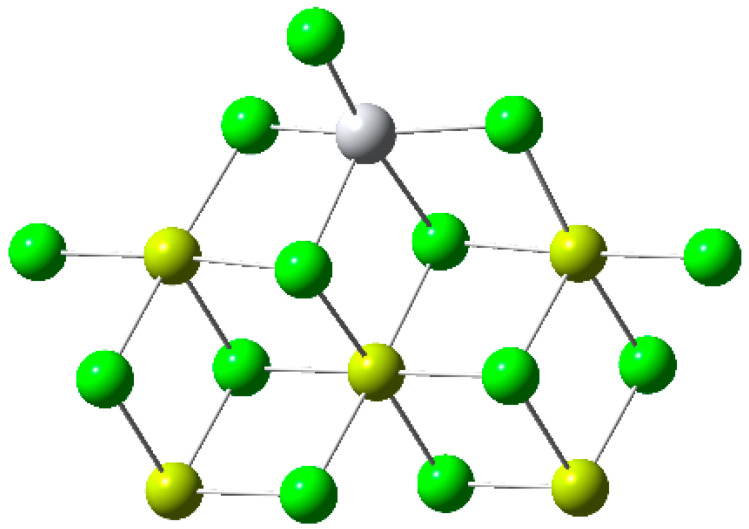
Size of the ZN catalyst used to evaluate the inhibition mechanisms. Note: Color code: Ti = light gray, Mg = yellow, Cl = green.

A TiCl_4_·(MgCl_2_)_14_ nanoplane (≈55 atoms) was used to model the active surface; it is the smallest cluster capable of reproducing Ti’s octahedral coordination and the correct density of Lewis sites (Figure 2). Recent DFT and machine-learning studies have shown that nanoplates containing 6–19 MgCl_2_ units are the most energetically accessible structures and thus realistic models of the essential Ziegler–Natta building blocks. This catalyst size offers an optimal trade-off between structural fidelity and computational cost, allowing stacking interactions and the Ti^4+^ arrangement to be captured without introducing periodic artefacts [34].

Finally, to evaluate the electrophilicity of the optimized complexes, the electrophilicity index was calculated according to the definition proposed by Parr et al. [35]:

(1) $$w=\frac{\mu^{2}}{2\eta }$$

In this context, the chemical potential (*μ*) and the molecular hardness (*η*) are fundamental parameters. The conceptual DFT framework [36] determines these values for a system with N electrons and a total electron energy E under a specific external potential. Specifically, *μ* and *η* are obtained by calculating the first and second derivatives of the energy, respectively. *μ* and *η* are estimated by finite differences and Koopman’s theorem [37].

(2) $$\mu =-\chi =\frac{1}{2}\left(\varepsilon_{L}+\varepsilon_{H}\right)\;\eta =\varepsilon_{L}-\varepsilon_{H}$$

The adsorption energies of the inhibitors were calculated using the following formula:

(3) $$\Delta E=E_{ADS}=E_{Com}-(E_{Inh}+E_{Cat})$$

where *E*_ADS_ is the adsorption energy, *E*_Com_ is the energy of the complex formed between the poison and the Ziegler–Natta (ZN) catalyst, *E*_Inh_ represents the energy of the isolated inhibitor species, and *E*_Cat_ is the energy of the catalyst in its canonical form.

To address the analysis of molecular reactivity, it is essential to understand how variations in the number of electrons affect the electronic density in a specific system. An effective approach for this purpose is the application of the Fukui function, which provides a powerful tool for evaluating how the electronic density, ρ(r), changes in response to modifications in the number of electrons, (N). This method takes into account the impact of a uniform and stable external potential, ν(r), and allows for the calculation of changes in electronic density using the first derivative of the system’s total energy, (E).

The Fukui function is defined by the following equation:

(4) $$f\left(r\right)={\left[\frac{\partial \rho (r)}{\partial N}\right]}_{\nu (r)}={\left[\frac{\delta \mu }{\delta \nu (r)}\right]}_{N}$$

This analysis is crucial for identifying areas within a molecule that are prone to participate in chemical reactions, as it allows for the differentiation of regions with high or low electronic density, which are indicative of susceptibility to nucleophilic, electrophilic, and radical attacks. In the present study, energy calculations were performed at a single point using density functional theory (DFT) with the B3LYP-D3 functional and the 6-311++G(d,p) basis set to apply this technique and obtain precise local descriptors.

#### Determination of Adsorption Energies in Solvent (n-Hexane)

In addition to the gas-phase stage, adsorption energies (*E*_ADS_) were evaluated in n-hexane using the implicit-solvation SMD approach. n-Hexane was selected because it is the conventional diluent in slurry-phase polymerization processes where the ZN catalyst is employed industrially; numerous practical studies describe the reaction in hexane media to ensure efficient mass transfer and a low-polarity environment [38]. The SMD model was chosen for its versatility and its specific parametrization for non-polar organic solvents, enabling reproduction of solvation free energies for neutral molecules with an average error below 1 kcal mol^−1^, and thus permitting direct comparison with gas-phase values [39].

### 2.2. Experimental Study

A laboratory-scale machine with temperature and pressure controllers (Parr Instrument Company, USA) was used to polymerize propylene in the gas phase in a nitrogen-purged fluidized-bed reactor at 70 °C and 27 bar. A fourth-generation Ziegler–Natta catalyst on MgCl_2_ (Sudchemie, Germany) was fed at a rate of 5 kg h^−1^ and activated on-site with triethylaluminum (TEAL, 98%, diluted in n-heptane, Merck, Germany). Shazand Petrochemical in Arak, Iran, sent polymer-grade propylene (1.2 t h^−1^) to the plant, and Merck in Germany sent hydrogen (30 g h^−1^, 99.99%) to manage the molecular weight. We controlled isotacticity by adding cyclohexyl methyl dimethoxysilane (CMDS, Merck, Germany) at a rate of 1 mol h^−1^. We used mass flow controllers (Brooks Instruments, USA) to add analytical grade dimethylamine (DMA) and diethylamine (DEA) to the monomer stream at levels between 0 and 140 ppm (DMA) and 0 and 170 ppm (DEA). Before sampling, the reactor was held at steady-state conditions for 45 min. After that, data on polymer productivity were recorded (Table 1).

### 2.3. Infrared Spectroscopy

Infrared (IR) analyses were carried out using a Nicolet iS50 FT-IR spectrometer from Thermo Fisher Scientific, purchased through their distributor in Madrid, Spain. The attenuated total reflection (ATR) mode was employed. Spectra were recorded at a resolution of 4 cm^−1^ over the 400–4000 cm^−1^ range, enabling highly sensitive and precise identification of the various absorption bands.

## 3. Results

### 3.1. Loss of Productivity in Polypropylene Production

To assess the ability of amines to halt the activity of the Ziegler–Natta (ZN) catalyst, the propylene polymerization reaction was investigated in a reactor. Controlled amounts of dimethylamine (0–140 ppm) and diethylamine (0–170 ppm) were introduced into a propane bed, followed by hydrogen, co-catalyst, and catalyst. The system was maintained at 70 °C and constant pressure for 45 min, allowing measurement of the decrease in productivity as a function of each amine’s concentration.

**Figure 3:**
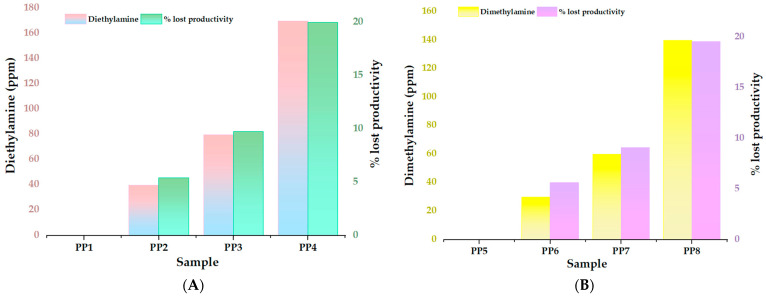
(**A**) Productivity loss in the polymerization process in the presence of diethylamine. (**B**) Productivity loss in the polymerization process in the presence of dimethylamine.

Figure 3 reveals striking results when comparing the influence of diethylamine and dimethylamine on ZN-catalyst efficiency in polypropylene manufacturing. Even though diethylamine levels are much higher than those of dimethylamine, both amines markedly affect productivity. Diethylamine peaks at 170 ppm, causing a 20% drop in productivity, whereas dimethylamine at 140 ppm leads to a 19.57% decline. This pattern suggests that dimethylamine may be more effective at hindering catalytic activity—possibly because its smaller size or stronger binding to active sites enables more efficient poisoning. By contrast, diethylamine, despite being present at higher concentrations, could be limited by steric hindrance or weaker binding, lessening its comparative impact.

### 3.2. Structural Characteristics of the Studied Inhibitors

It is essential to understand the intrinsic properties of dimethylamine (DMA) and diethylamine (DEA) before assessing their behavior with the catalytic system, as these characteristics underpin their inhibition mechanisms. In this context, the HOMO–LUMO gap, chemical potential (μ), and hardness (η) are classical descriptors of electronic stability and resistance to excitation—concepts established by Parr et al. as fundamental to molecular reactivity [40,41]. Qualitatively, these values confirm that both amines possess a highly resilient electronic “core”: the HOMO–LUMO gap (~136 kcal·mol^−1^) indicates a low tendency to lose or gain electrons, while μ (≈73.8 kcal·mol^−1^) and η (≈136 kcal·mol^−1^) demonstrate analogous behavior under electronic perturbations (Table 2).

Quantitatively, however, the differences in these parameters (<1 kcal·mol^−1^) fall below the typical uncertainty of DFT calculations (±1–2 kcal·mol^−1^), rendering them unreliable for distinguishing the relative stability of DMA and DEA.

**Figure 4:**
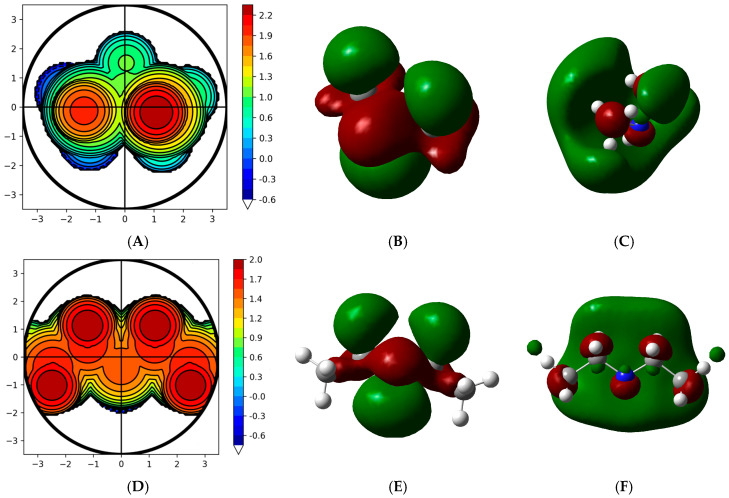
Steric topographic map of DMA (**A**), highest occupied molecular orbital diagram of DMA (**B**), lowest unoccupied molecular orbital diagram of DMA (**C**), steric topographic map of DEA (**D**), highest occupied molecular orbital diagram of DEA (**E**), and lowest unoccupied molecular orbital diagram of DEA (**F**). In the maps, the nitrogen atom is located at the origin. Note: Color code: Subfigures B, C, E, and F (HOMO/LUMO): The green and red surfaces represent the positive (+) and negative (−) phases of the wave function. H = white, C = dark gray, Al = pink, N = blue.

Instead, we examined two-dimensional steric topographic maps (Figure 4A–D) generated with UCSF ChimeraX v1.8. The buried volume (%VBur)—40.2% for DMA versus 51.4% for DEA—exceeds the ±2% error margin and conclusively demonstrates that DEA imposes a more substantial steric blockade, hindering access to the catalyst’s active site. Although the electrophilicity difference (Δω ≈ 0.19 kcal·mol^−1^) lies below DFT’s precision threshold, its agreement with the buried volume provides valuable qualitative support, indicating that DMA, with its lower steric congestion, can accept electrons more readily and coordinate more efficiently [31].

#### Local Descriptors of the Amines

Reactivity analyses were conducted using UKA FOKUI 2.00 software to examine the reactivity of the amines as catalyst poisons. The findings, along with the atomic numbering for each chemical species, are presented in Table 3 and Table 4.

Table 3 shows the local descriptors for dimethylamine, indicating that the nitrogen atom (1) is the most susceptible to nucleophilic attacks, as evidenced by its high *f^−^* value (0.7475). In contrast, the adjacent carbon atoms (atoms 2 and 6) exhibit the highest *f^+^* values (0.3626), suggesting their predisposition to electrophilic attacks. These results imply that the electronegativity of the nitrogen increases the positive charge density on the adjacent carbons, enhancing their reactivity towards electrophilic species. On the other hand, the secondary carbon atoms (5 and 9) show moderate reactivity towards nucleophilic and radical attacks, as indicated by their intermediate *f^−^* and *f*^0^ values. In contrast, the remaining carbon and hydrogen atoms exhibit low *f^+^* and *f^−^* values, indicating minimal susceptibility to nucleophilic and electrophilic interactions. Thus, the analysis highlights that the chemical reactivity of dimethylamine is primarily concentrated on the nitrogen and the adjacent carbons, which are the most reactive sites in the molecule.

The analysis of local descriptors *f^+^*, *f^−^*, and *f*^0^ for diethylamine reveals that the nitrogen atom (Atom 1) is the most significant reactive center, with an *f*^−^ value of 0.6749, indicating a strong nucleophilic tendency, and an *f*^0^ value of 0.3455, suggesting moderate susceptibility to radical attacks. The carbons 2 and 5, which are directly bonded to the nitrogen, show the highest *f*^+^ values (0.1013 and 0.1010, respectively), denoting a greater predisposition to electrophilic attacks at these sites. Although these carbons also exhibit low *f*^−^ values, they are the second most likely sites for nucleophilic interactions after the nitrogen.

On the other hand, the carbons in the methyl and ethyl groups (atoms 10–16) show low values across all descriptors, indicating reduced reactivity compared to the central amine group. However, some propensity for radical interactions is observed in carbons 9 and 13, as evidenced by their relatively higher *f*^0^ values. Overall, these results highlight that the reactivity of diethylamine is primarily concentrated at the nitrogen atom and the adjacent carbons, with a clear differentiation in the propensity for nucleophilic and electrophilic attacks (Table 4).

### 3.3. Interaction Direct Amine Adsorption on Ziegler–Natta Active Sites

In this section, we analyzed five mechanistic pathways—from the direct binding of the amines to TiCl_4_ through chloride elimination and HCl release—in order to explore every possible route by which the catalyst might be affected (Diagram 2).

**Scheme 1:**
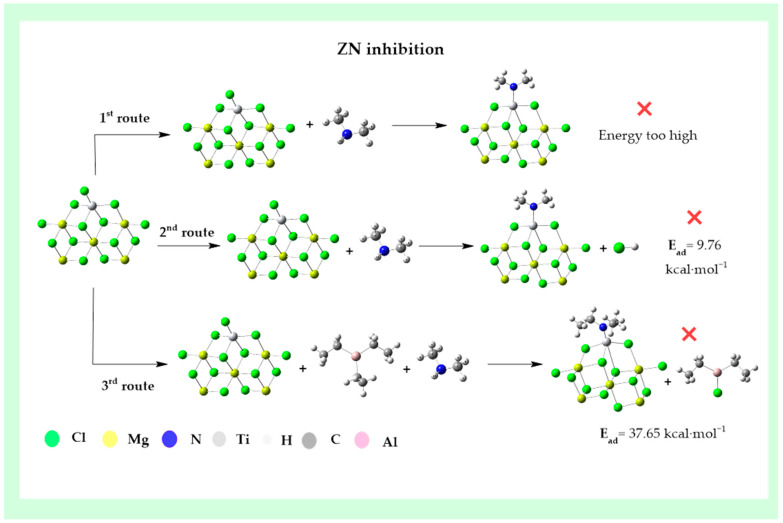
Computationally evaluated pathways for predicting smine-induced inhibition of the ZN vatalyst.

Scheme 1 outlines three initial scenarios explored to understand how amines might adversely affect ZN. In the first pathway, the amine attempts to bind directly to Ti, but the associated energy barrier is prohibitively high, rendering the route unfeasible. The second pathway envisions substitution of a chloride after amine approach; however, adsorption is endothermic (activation energy = +9.76 kcal mol^−1^), signaling low affinity and, likewise, ruling it out. The third pathway proposes that TEAL first activates the catalyst, the amine subsequently binds, and AlEt_2_Cl is released—but the energy cost is even higher (activation energy = +37.65 kcal mol^−1^), confirming that the TEAL-facilitated route under these conditions also fails to block the active site. Given the energetic impracticality of these initial pathways, the study shifted to examining amine–TEAL interactions to form a complex that could then bind to the ZN catalyst (Section 3.4 and Section 3.5).

### 3.4. Mechanism Between Amines and TEAL: Energetic Analysis

After ruling out the thermodynamic possibility that amines interact directly with the ZN catalyst, we investigated an alternative pathway involving the prior coordination of the amines with the co-catalyst TEAL. This compound, known for its role as an alkylating and reducing agent in the catalytic system, can also scavenge and remove impurities present in the reaction mixture [42]. Therefore, the optimized structures, energies, and activation barriers of the complexes formed between dimethylamine (DMA) and diethylamine (DEA) with TEAL were analyzed computationally, thereby evaluating their stability and potential to act as intermediates in the deactivation process.

#### Complex Formation Between Toxic Agents and the Co-Catalyst

**Scheme 2:**
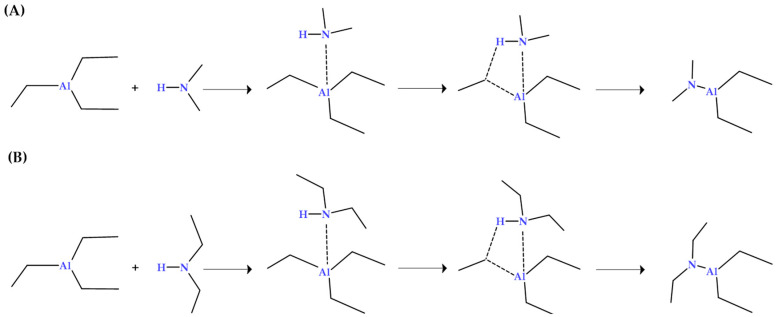
Interaction of amines with TEAL; (**A**) Interaction between TEAL and DMA; (**B**) Interaction between TEAL and DEA.

To investigate the inhibitory effect of the amines on the catalytic system, a study was conducted on the formation of complexes between TEAL and DMA and DEA (see Scheme 2). The evaluation considered both thermodynamic and kinetic aspects, using values of Gibbs free energy (ΔG), forward (Ea^+^) and reverse (Ea^−^) activation barriers, as well as the equilibrium constant (K), all calculated using DFT.

The equilibrium constant K is related to the change in Gibbs free energy between reactants and products at constant temperature. A high K value (much greater than 1) suggests that, at equilibrium, the reaction strongly favors product formation. Conversely, a low K value (much less than 1) indicates that the equilibrium position lies toward the reactants, even if the products are thermodynamically stable—an outcome that may be influenced by high kinetic barriers. The values of ${E_{a}}^{+}$, ${E_{a}}^{-}$, and ΔG were calculated from the Gibbs free energies of each species, optimized using the following expressions:

(5) $${E_{a}}^{+}=G_{TS}-G_{Reactants}$$

(6) $${E_{a}}^{-}=G_{TS}-G_{Products}$$

(7) $$∆G=G_{Products}-G_{Reactants}$$

(8) $$K=\exp\left(-\frac{∆G}{RT}\right)$$

Table 5 presents the results of evaluating TEAL’s ability to form complexes with these species.

**Figure 5:**
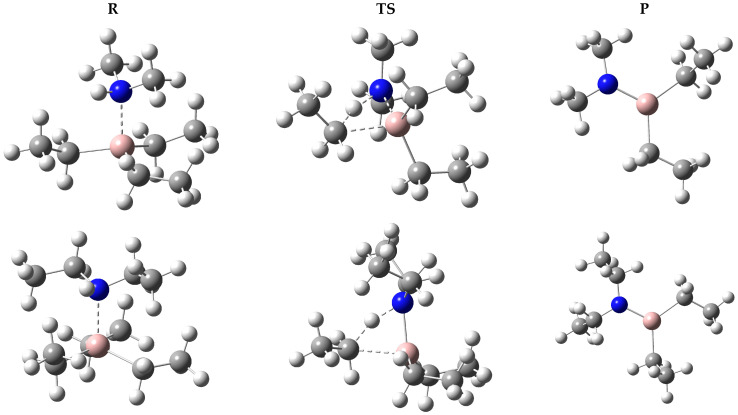
Interaction between the amines and the cocatalyst. R = reactants; TS = transition State; P = products. Note: Color code: H = white, C = dark gray, Al = pink, N = blue.

Figure 5 displays the optimized structures of the reactants, transition states, and products resulting from the interactions between TEAL and the amines (dimethylamine and diethylamine).

**Figure 6:**
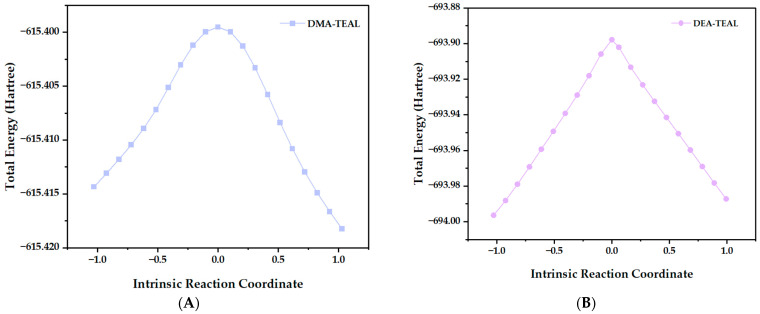
IRC for the DMA–TEAL transition-state complex (**A**); IRC for the DEA–TEAL transition-state complex (**B**).

From a thermodynamic perspective, both processes exhibit a negative ΔG, indicating that complex formation occurs spontaneously under standard conditions. However, kinetic analysis reveals a notable difference: the formation of the TEAL–DMA complex has a moderate activation barrier (27.11 kcal·mol^−1^), allowing it to proceed at room temperature. In contrast, the TEAL–DEA complex presents a much higher barrier (126.05 kcal·mol^−1^), significantly limiting its formation without additional energy input. The high equilibrium constants obtained (K ≈ 10^13^–10^15^) suggest that, once the activation barriers are overcome, the conversion to products is highly favorable. Nevertheless, the kinetics hinder the formation of the DEA complex due to its steric hindrance and an unfavorable geometry for coordination with the aluminum center. This behavior is clearly observed in the reaction profiles (Figure 6), where the IRC curves show a smoother energy transition for DMA compared to DEA.

### 3.5. Adsorption of the Pre-Formed TEAL–Amine Complex on ZN Catalytic Centers

Building on the favorable results obtained for the DMA–TEAL and DEA–TEAL complexes, we analyzed how these complexes interact with the ZN catalyst. The goal was to establish whether prior amine capture by TEAL affects the energetic stability of the catalytic system and the amines’ ability to deactivate the catalyst’s active center. To that end, we calculated the adsorption energies associated with the interaction between each TEAL–amine complex and the active TiCl_4_/MgCl_2_ surface, and compared them with the pathways examined previously.

**Figure 7:**
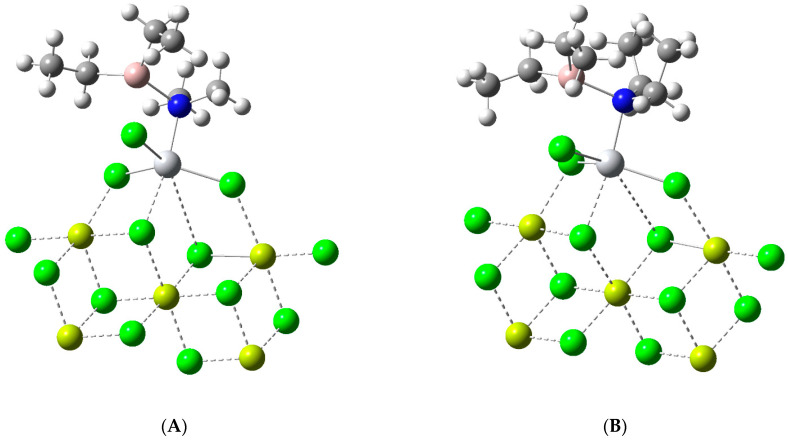
(**A**) Optimized structure of the ZN–TEAL–DMA system; (**B**) Optimized structure of the ZN–TEAL–DEA system. Note: Color code: H = white, C = dark gray, Al = pink, N = blue, Ti = light gray, Mg = yellow, Cl = green. Dashed lines outline the MgCl_2_ slab used as support.

Figure 7 depicts the optimized configurations of the species arising from the interaction of the ZN catalyst, the TEAL co-catalyst, and the amines DMA and DEA. In both images, the amine coordinates to the aluminum atom of TEAL, which is itself bound to the ZN active region, represented by a TiCl_4_/MgCl_2_ monocluster.

The arrangement containing DMA is more compact and experiences less steric hindrance (Figure 7A), promoting a strong, stable interaction. In contrast, the DEA arrangement exhibits greater steric congestion owing to its bulkier ethyl groups, which could hamper both the formation and the stability of the complex (Figure 7B).

From the data in Table 6, the ZN–TEAL–DMA system exhibits an adsorption energy (E_a_d) of −22.9 kcal mol^−1^ in the gas phase and −25.4 kcal mol^−1^ in n-hexane, compared with −18.2 kcal mol^−1^ and −14.9 kcal mol^−1^ for ZN–TEAL–DEA. These energy values indicate that DMA, owing to its lower steric hindrance, binds 5–10 kcal mol^−1^ more strongly than DEA, resulting in more effective inhibition of the catalytic site. A non-polar solvent further enhances DMA’s binding affinity while reducing that of DEA, as confirmed by experimental data in which DMA-type amines must be removed to avoid productivity losses. Overall, the energies point to moderate, reversible adsorption within the −15 to −25 kcal mol^−1^ range.

These energy differences explain why, at comparable concentrations, DMA causes nearly the same productivity loss as DEA—even though less DMA is required: the greater energy released when the DMA-TEAL complex forms intensifies the damage. Finally, because the enthalpy (H) and Gibbs free energy (G) of the ZN–(TEAL·DMA) system are lower than those of free ZN, adsorption is thermodynamically feasible. Paradoxically, this overall stabilization locks the active site and prevents proper catalytic action.

### 3.6. Spectroscopic Analysis

**Figure 8:**
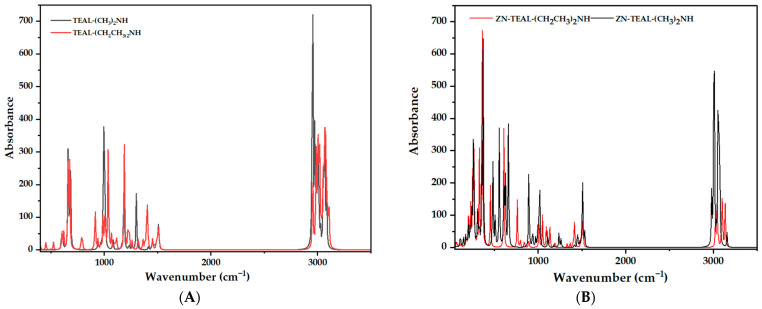
IR spectrum for the interaction between the amines and TEAL (**A**); IR spectrum for the interaction among the ZN catalyst, the amines, and TEAL (**B**).

In the IR spectra of Figure 8A, a weak band appears near 615 cm^−1^, assigned to the ν(Al–N) stretch characteristic of Al–N bonds in wurtzite phases—direct evidence of dimethylamine’s dative coordination [43,44]. The multiplet between 660 and 710 cm^−1^ comprises (i) rocking of Al–CH_3_ fragments produced after trialkylaluminum exposure [45,46] (ii) the out-of-plane N–H wag typical of secondary amines (~730 cm^−1^), and (iii) CH_2_ rocking from long ethyl chains (720 ± 10 cm^−1^), confirming retention of the ethyl groups and preservation of the nitrogen proton [47,48]. The most intense peak in the region (~1000 cm^−1^) is attributed to in-phase CH_3_ rocking, with contributions from ν(Al–C) in the Et–Al framework, reflecting the added rigidity imposed by coordination. Peaks at 1200–1190 cm^−1^ correspond to C–N stretching, shifted about +15 cm^−1^ relative to free dimethylamine because of Al←N electron redistribution.

To concisely summarize the main bands identified and their corresponding vibrations, Table 7 presents (i) the wavenumber range, (ii) the predominant band, (iii) the spectral assignment, (iv) the shift relative to the free molecules, and (v) the structural or chemical implication of each peak.

The infrared spectra corroborate the proposed deactivation pathway. When these compounds (DMA and DEA) are brought into contact with the ZN catalyst, the Al–N signal persists, while the bands associated with Al–C/Ti–C bonds (500–900 cm^−1^) drop by more than 30%. At the same time, the ν(C–H) stretches of the ethyl groups attached to Al increase, indicating that alkyl transfer from Al to Ti is strongly hindered. The intensity ratio of ν(Al–N) to ν(Al–C) is higher for DMA than for DEA, aligning with the larger productivity reduction seen in the experimental data.

## 4. Discussion

There are few studies on the poisoning of Ziegler–Natta (ZN) catalysts in the presence of amines, with one of the most detailed being that of Sacchi et al. (1988), which analyzes the role of amines as additives in ZN catalytic systems for polypropylene polymerization [6]. The research focuses on two amines, ethyl-tri-n-butylamine (Et_3_N) and 2,2,6,6-tetramethylpiperidine (TMPip), and their influence on the productivity and selectivity of catalysts based on AlEt_3_ and ZnEt_2_. One of the most significant findings is the identification of the dual effects that amines have on the catalyst’s active sites. On one hand, amines are observed to poison both isotactic and atactic sites, which is attributed to the interaction between the non-complexed base and the active sites on the catalytic surface. On the other hand, amines also have the ability to activate isotactic sites, which is related to the presence of metal–alkyl complexes with the base in solution. The selectivity of TMPip in poisoning atactic centers, due to its greater steric hindrance, underscores the importance of the amine’s structure in catalytic interactions. Additionally, a difference in productivity was observed between AlEt_3_ and ZnEt_2_-based catalysts, where the addition of a small amount of base activated AlEt_3_ catalysts, while in ZnEt_2_ systems, a minimal productivity was seen initially, followed by a significant increase with higher base concentrations.

Mehrdad Fallah et al. investigated the coordination of CO_2_, O_2_, NH_3_, CH_3_OH, and H_2_O molecules with organoaluminum compounds (TMA, TIBAL, DMAC) [42]. The study reveals that the interaction between amines, specifically ammonia (NH_3_), and organometallic aluminum cocatalysts is significant, as amines tend to form stable complexes with the cocatalysts, leading to the deactivation of the catalyst’s active sites. The energy of complexation of NH_3_ with the cocatalysts was found to be higher compared to other impurities, indicating that amines have a greater adsorption capacity. This phenomenon can inhibit polymerization and negatively affect the final polymer’s properties, such as isotacticity and molecular weight. Additionally, the study suggests that adsorption is more favorable at monomeric Al centers compared to dimeric forms, highlighting the importance of considering the purity of reactants and the reaction medium to optimize polymerization efficiency.

The computational findings of this study indicate that the initial mechanisms proposed, in which reactions occur directly with the main catalyst, are not thermodynamically viable. This underscores the importance of considering the intrinsic limitations of theoretical models, as the idealized conditions assumed in the simulations—such as the gas phase and the absence of solvent effects—may not accurately represent real factors like reactor pressure and temperature, which directly influence activation energy and the stability of intermediate complexes. These simplifications could lead to an overestimation of energy values and a misinterpretation of the process feasibility. However, in the mechanism where the interaction first occurs between the co-catalyst (TEAL) and the amines, the findings indicate thermodynamic viability in agreement with previous studies, such as that conducted by Mehrdad Fallah et al. [42]. This discovery suggests that the co-catalyst plays a crucial role in stabilizing intermediate complexes, facilitating the activation of the main catalyst, and providing a more favorable reaction pathway.

## 5. Conclusions

This combined theoretical–experimental study shows that the drop in ZN productivity is chiefly driven by the sequence amine → TEAL → amine·TEAL complex → adsorption on ZN. DFT calculations yield adsorption energies of –22.9 kcal mol^−1^ for dimethylamine (DMA) and –18.2 kcal mol^−1^ for diethylamine (DEA) in the gas phase, with marked contrasts in n-hexane (SMD model): the DMA interaction strengthens to –25.4 kcal mol^−1^, whereas the DEA interaction weakens to –14.9 kcal mol^−1^, confirming that the non-polar industrial medium favors TEAL·DMA formation and consequent active-site blocking. These computational trends align with the reactor experiments, where 140 ppm DMA and 170 ppm DEA reduced polypropylene productivity by 19.6% and 20%, respectively, showing that DMA achieves an equivalent detrimental effect at a lower concentration. Finally, IR spectroscopy supports the proposed mechanism: after contact with ZN, the ν(Al–N) band near 615 cm^−1^ persists, the Al–C/Ti–C bands (500–900 cm^−1^) drop by more than 30%, the N–H stretching frequency shifts from ~3300 to 3200 cm^−1^, and the ν(C–N) band rises by +15 cm^−1^.

## Figures and Tables

**Table 1:** Concentrations of the amines and names of the samples studied.

Materials	PP1	PP2	PP3	PP4	PP5	PP6	PP7	PP8
Catalyst, Kg/h	5	5	5	5	5	5	5	5
Propylene, TM/h	1.2	1.2	1.2	1.2	1.2	1.2	1.2	1.2
TEAl, Kg/h	0.25	0.25	0.25	0.25	0.25	0.25	0.25	0.25
Hydrogen, g/h	30	30	30	30	30	30	30	30
Dimethylamine	0	0	0	0	0	30	60	140
Diethylamine	0	40	80	170	0	0	0	0
Selectivity control agent, mol/h	1	1	1	1	1	1	1	1
T, °C	70	70	70	70	70	70	70	70
Pressure, bar	27	27	27	27	27	27	27	27
Productivity Ziegler–Natta (TM/Kg)	46	43.5	41.5	36.8	46	43.4	41.8	37
% productivity loss	0	5.43	9.78	20.00	0	5.65	9.13	19.57

Note: The assays were organized in two independent series: the first evaluated diethylamine (DEA) in PP2–PP4, with PP1 as blank; the second studied dimethylamine (DMA) in PP6–PP8, with PP5 as blank. The blanks, run without amine, fix the base productivity and ensure that the measured effects are attributed exclusively to the amine in each series, with no carryover between them.

**Table 2:** Structural and electronic properties of the analyzed amines, calculated using the B3LYP/6-311++G(d,p) theoretical approach.

	ΔE_HOMO–LUMO_ (kcal·mol^−1^)	µ (kcal·mol^−1^)	η (kcal·mol^−1^)	Electrophilicity (kcal·mol^−1^)	Natural Bond Orbital Charge on N	N–C Bond length (Å)	Buried Volume (%V_Bur_)
(CH_3_)_2_NH	136.12	73.90	136.12	20.06	−0.67260	1.457	40.2
(C_2_H_5_)_2_NH	136.39	73.64	136.39	19.87	−0.67926	1.525	51.4

**Table 3:** Local descriptors for dimethylamine.

Local Descriptors				
Atom	*f^+^*	*f^−^*	*f* ^0^	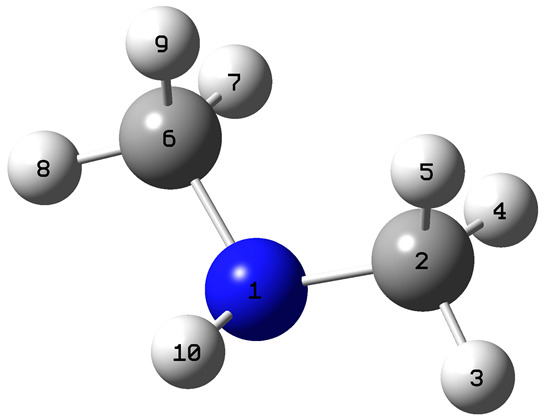
1	0.0189	0.7475	0.3832	
2	0.3626	0.0260	0.1943	
3	0.0357	0.0062	0.0210	
4	0.0288	0.0093	0.0190	
5	0.0463	0.0825	0.0644	
6	0.3625	0.0260	0.1942	
7	0.0288	0.0093	0.0190	
8	0.0357	0.0062	0.0210	
9	0.0463	0.0825	0.0644	
10	0.0344	0.0045	0.0195	

H = white, C = dark gray, N = blue.

**Table 4:** Local descriptors for diethylamine.

Local Descriptors				
Atom	*f^+^*	*f^−^*	*f* ^0^	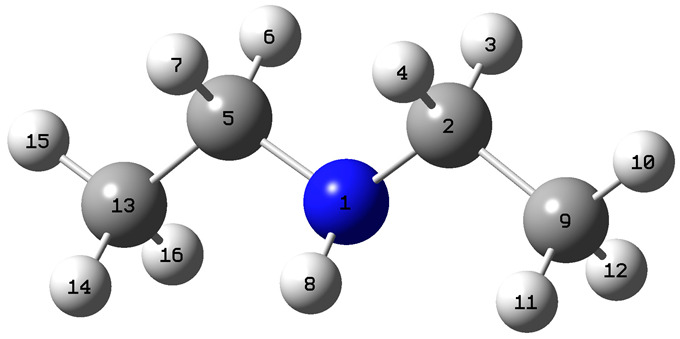
1	0.0160	0.6749	0.3455	
2	0.1013	0.0195	0.0604	
3	0.0201	0.0224	0.0212	
4	0.0368	0.0991	0.0680	
5	0.1010	0.0195	0.0602	
6	0.0201	0.0223	0.0212	
7	0.0369	0.0991	0.0680	
8	0.0057	0.0097	0.0077	
9	0.2365	0.0097	0.1231	
10	0.0157	0.0019	0.0088	
11	0.0607	0.0021	0.0314	
12	0.0181	0.0030	0.0106	
13	0.2364	0.0098	0.1231	
14	0.0606	0.0021	0.0314	
15	0.0157	0.0019	0.0088	
16	0.0182	0.0030	0.0106	

H = white, C = dark gray, N = blue.

**Table 5:** Absolute and relative energies (kcal mol^−1^) for the interaction between TEAL and toxic molecules, calculated at the B3LYP/6-311++G(d,p) level in the gas phase (see Diagram 2).

	E (kcal·mol^−1^)	H (kcal·mol^−1^)	G (kcal·mol^−1^)	E_a_^+^ (kcal·mol^−1^)	E_a_^−^ (kcal·mol^−1^)	∆G	*K*
TEAL-(CH_3_)_2_NH (R)	−386,200.17	−386,008.05	−386,047.27	27.11	−46.08	−18.97	5.79 × 10^13^
TEAL-(CH_3_)_2_NH (TS)	−386,169.11	−385,980.74	−386,020.16				
TEAL-(CH_3_)_2_N (PR)	−386,204.92	−386,014.50	−386,066.24				
TEAL-(C_2_H_5_)_2_NH (R)	−435,551.70	−435,322.31	−435,364.99	126.05	−147.33	−21.28	1.70 × 10^15^
TEAL-(C_2_H_5_)_2_NH (TS)	−435,423.13	−435,195.81	−435,238.94				
TEAL-(C_2_H_5_)_2_N (PR)	−435,557.73	−435,330.11	−435,386.27				

**Table 6:** Absolute energies for the interaction between the ZN catalyst and hazardous compounds calculated at the D3-B3LYP/6-311++G(d,p) level in the gas phase.

Gas Phase					n-Hexane SMD			
Structure	E (kcal·mol^−1^)	H (kcal·mol^−1^)	G (kcal·mol^−1^)	Ead (kcal·mol^−1^)	E (kcal·mol^−1^)	H (kcal·mol^−1^)	G (kcal·mol^−1^)	Ead (kcal·mol^−1^)
ZN	−4,915,906.72	–4,915,896.61	–4,915,932.13	–22.94	−4,915,957.88	−4,915,947.78	−4,915,982.78	−25.37
TEAL-(CH_3_)_2_N	–336,094.15	–335,953.14	–335,987.59		−336,097.67	−335,956.79	−335,990.99	
ZN-(CH_3_)_2_N-TEAL	–5,252,023.80	–5,251,871.47	–5,251,925.93		−5,252,080.92	−5,251,928.06	−5,251,982.74	
ZN-(C_2_H_5_)_2_N-TEAL	–5,301,371.90	–5,301,182.29	–5,301,239.84	–18.22	−5,301,424.39	−5,301,322.20	−5,301,366.84	−14.95
TEAL-(C_2_H_5_)_2_N	–385,446.96	–385,268.74	–385,307.63		−385,451.57	−385,273.48	−385,311.98	

**Table 7:** Assigned IR vibrations and spectroscopic shifts for the TEAL·(CH_3_)_2_NH complex.

Region (cm^−1^)	Main Band	Vibrational Assignment	Shift vs. Free Species	Chemical/Structural Significance
3100–2800 (max ≈ 2960)	Set of very intense peaks	V_as_ and ν_s_. C–H stretches of CH_3_/CH_2_ in TEAL (ethyl groups)	Positions essentially unchanged (<5 cm^−1^); intensity increases owing to the large number of hydrogens	Confirms that the alkyl groups are retained after coordination
≈3260 → 3200	Moderate peak (often overlapping)	N–H stretch	Downshift of ~30–40 cm^−1^ relative to free DMA (≈3300 cm^−1^)	Donation of the N lone pair to Al weakens N–H, lowering its frequency → direct evidence of Al←N coordination
1500–1450	Medium doublet	CH_2_ scissoring and asymmetric CH_3_ deformation	Slight shifts (≤10 cm^−1^)	Sensitive to minor conformational changes of the ethyl group upon binding to tetracoordinate Al
≈1380 and 1360	Sharp bands	Symmetric CH_3_ deformation and N–H “umbrella”	N–H band shows slight intensity decrease	Supports a weaker N–H environment after coordination
1150–1050 (max ≈ 1110)	Medium band	C–N stretch	Shift to higher energy (≈+15 cm^−1^) vs. free DMA	Higher frequency indicates greater partial π-character in C–N due to back-donation to coordinated N (less σ-density, more π-acceptance)
≈950–850	Group of peaks	CH_3_–CH_2_ rocking/rotation	—	Reflects added rigidity of the ethyl backbone once bound to Al
≈720–640	New medium band	ν(Al–C)ethyl + CH_2_ wag components	—	First evidence that ethyl groups remain tetrahedrally bound to Al

## Data Availability

The original contributions presented in this study are included in the article. Further inquiries can be directed to the corresponding author.
